# Experimental Non-Violation of the Bell Inequality

**DOI:** 10.3390/e20050356

**Published:** 2018-05-10

**Authors:** Tim N. Palmer

**Affiliations:** Department of Physics, University of Oxford, Oxford OX1 3PU, UK; tim.palmer@physics.ox.ac.uk

**Keywords:** Bell theorem, fractal geometry, *p*-adic metric, singular limit, gravity, conspiracy, free will, number theory, quantum potential

## Abstract

A finite non-classical framework for qubit physics is described that challenges the conclusion that the Bell Inequality has been shown to have been violated experimentally, even approximately. This framework postulates the primacy of a fractal-like ‘invariant set’ geometry $I_{U}$ in cosmological state space, on which the universe evolves deterministically and causally, and from which space-time and the laws of physics in space-time are emergent. Consistent with the assumed primacy of $I_{U}$, a non-Euclidean (and hence non-classical) metric $g_{p}$ is defined in cosmological state space. Here, *p* is a large but finite integer (whose inverse may reflect the weakness of gravity). Points that do not lie on $I_{U}$ are necessarily $g_{p}$-distant from points that do. $g_{p}$ is related to the *p*-adic metric of number theory. Using number-theoretic properties of spherical triangles, the Clauser-Horne-Shimony-Holt (CHSH) inequality, whose violation would rule out local realism, is shown to be undefined in this framework. Moreover, the CHSH-like inequalities violated experimentally are shown to be $g_{p}$-distant from the CHSH inequality. This result fails in the singular limit p=∞, at which $g_{p}$ is Euclidean and the corresponding model classical. Although Invariant Set Theory is deterministic and locally causal, it is not conspiratorial and does not compromise experimenter free will. The relationship between Invariant Set Theory, Bohmian Theory, The Cellular Automaton Interpretation of Quantum Theory and *p*-adic Quantum Theory is discussed.

## 1. Introduction

Recent experiments (e.g., [1]) have seemingly put beyond doubt the conclusion that the CHSH version

(1) |Corr(0,0)+Corr(1,0)+Corr(0,1)−Corr(1,1)|≤2

of the Bell Inequality is violated robustly for a range of experimental protocols and measurement settings. As a result, it is very widely believed that physical theory cannot be based on Einsteinian notions of realism and local causality (‘local realism’). Here, Corr(X,Y) denotes the correlation between spin measurements performed by Alice and Bob on entangled particle pairs produced in the singlet quantum state, where X=0,1 and Y=0,1 correspond to pairs of freely-chosen points on Alice and Bob’s celestial spheres, respectively.

Of course, in the *precise* form as written, (1) has not been shown to have been violated experimentally. In practice, the four correlations on the left-hand side of (1) are each estimated from a separate sub-ensemble of particles with measurements performed at different times and/or spatial locations. Hence, for example, the measurement orientation corresponding to Y=0 for the first sub-ensemble cannot correspond to *precisely* the same measurement orientation Y=0 for the second sub-ensemble; as a matter of principle, Bob cannot shield his apparatus from the effects of ubiquitous gravitational waves associated for example with distant astrophysical events. Hence, as a matter of principle, what is actually violated experimentally is not (1) but

(2) $$|\mathrm{Corr}\left(0,0\right)+\mathrm{Corr}\left(1,0^{\prime }\right)+\mathrm{Corr}\left(0^{\prime },1\right)-\mathrm{Corr}\left(1^{\prime },1^{\prime }\right)|\leq 2,$$

where, relative to the Euclidean metric of space-time, $0\approx 0^{\prime }$ and $1\approx 1^{\prime }$ for *X* and *Y*.

Could the difference between 0 ≈ 0’, $1\approx 1^{\prime }$ on the one hand, and $0=0^{\prime }$, $1=1^{\prime }$ on the other, actually matter? More specifically, is there a plausible framework for physical theory where (1) is the singular [2] rather than the smooth limit of (2) as $0^{\prime }\to 0$, $1^{\prime }\to 1$ and, therefore, where (2) is in some sense physically distinct from (1), no matter how accurate are our finite-precision experiments? Intuitively, it would seem not, as Bell [3] himself argued with a form of ‘epsilonic’ analysis. Indeed, common sense might suggest to even contemplate such a possibility would be to entertain a theory that was not only grotesquely fine-tuned, but one that was inconsistent with the fact that the experimental violation of (2) does not require any precision in setting the polariser orientations.

The purpose of this paper is to argue that, in this respect, we are being fooled by our intuition. It is worthwhile beginning the discussion with a close analogy: the Penrose Impossible Triangle (sometimes known as the tribar). The triangle seems impossible because we intuitively assume that any two sides of the triangle necessarily become *close* at a common vertex. Relaxing this metric assumption makes it possible to construct such Penrose Triangles in 3D physical space: it is the projection into 2D of such a 3D structure that provides the illusion (but not the reality) of inconsistency.

The relevance of this example is to draw attention to the notion of distance. There is no doubt that space-time has a locally Euclidean metric. However, should we assume such a metric for state space? In conventional quantum theory based on complex Hilbert Space, this assumption is forced on us. However, motivated by both nonlinear dynamical systems theory and *p*-adic number theory, we outline in Section 2 a plausible and robust locally causal framework where the metric on state space is explicitly not Euclidean. This framework arises from the ‘invariant set’ postulate [4,5,6] that a certain fractal-like subset $I_{U}$ of cosmological state space is primal in the sense that the universe itself can be considered a deterministic dynamical system evolving on $I_{U}$, and moreover that space-time and the laws of physics in space-time are emergent from the geometry of $I_{U}$. Within ‘Invariant Set Theory’, complex Hilbert states have finite frequentist probabilistic interpretations as incompletely defined trajectory segments on $I_{U}$, requiring squared amplitudes and complex phases take rational values. By implication, complex Hilbert states with irrational squared amplitudes or irrational complex phases have no status as probabilistically defined trajectory segments on $I_{U}$ and are therefore ’non-ontic’. A key number theorem is introduced that establishes an incompatibility between rational angles and rational cosines and which completely underpins the viability of the invariant set postulate as a realistic causal basis for quantum physics. In Section 2, a metric $g_{p}$ (where *p* is a large integer) is introduced on state space, which respects the fundamental primacy of $I_{U}$ and with respect to which ontic and non-ontic Hilbert states are necessarily distant from one another.

After a warm-up discussion in Section 3 where the number theorem above is used to account for the non-commutativity of spin observables in Invariant Set Theory, in Section 4 we discuss the Bell Theorem. It is shown that the violation of (2) is generically robust to $g_{p}$-small-amplitude perturbations. However, the set of all inequalities encompassed by such perturbations does not and cannot include the Bell inequality (1) itself, whose violation would be needed to rule out local realism [7]. As shown, (1) is necessarily constructed from Hilbert states with irrational descriptors, i.e., non-ontic states not lying on $I_{U}$ and therefore $g_{p}$ distant from the ontic states lying on $I_{U}$. In this sense, (1) is neither satisfied nor violated in Invariant Set Theory: it is simply undefined. This is not so much a loophole as a gaping chasm in the Bell Theorem, allowing a new type of a locally causal theory as a candidate descriptor of quantum physics (and hence, potentially, a novel approach to synthesise quantum and gravitational physics). Invariant Set Theory has the added bonus that it is essentially a finite theory, in contradistinction with quantum theory, where the role of the infinitesimal appears to be foundational [8]. As discussed in Section 5, although deterministic, Invariant Set Theory is not conspiratorial and respects experimenter free will. In Section 6, Invariant Set Theory is compared with Bohmian Theory, ’tHooft’s Cellular Automaton Interpretation of Quantum Theory and *p*-Adic Quantum Theory. Further discussion and analysis of the issues of robustness and local causality are provided in Section 7.

## 2. Invariant Set Theory

**Figure 1:**
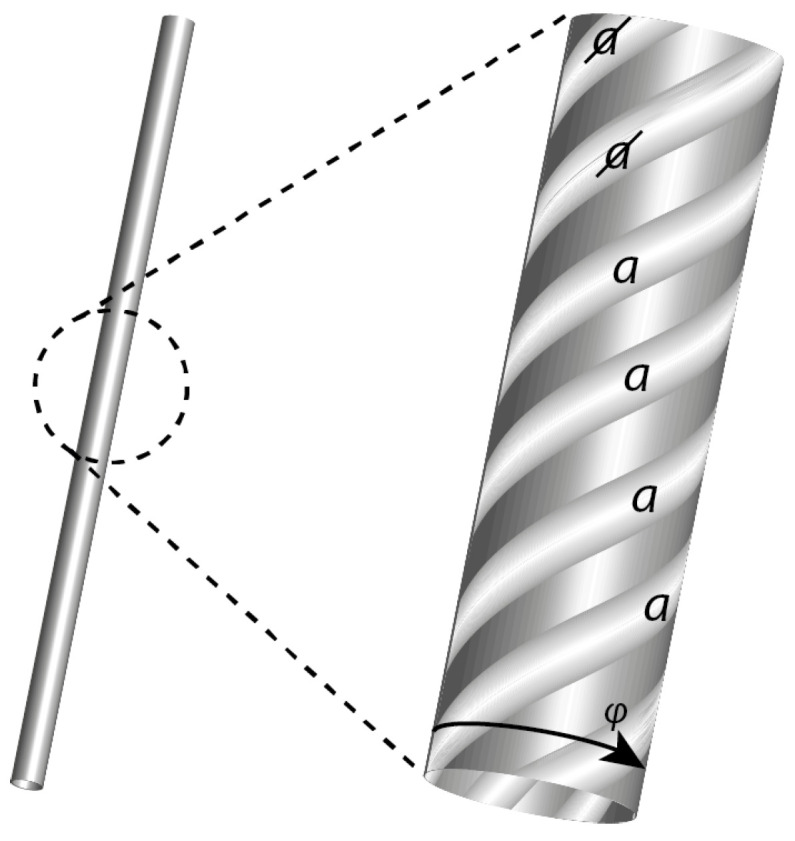
A state-space trajectory segment, which appears to be a simple line on some coarse scale, is in fact found to be, on magnification, a helix of trajectories. On further magnification, each of these helical trajectory segments is itself a helix of trajectories, and so on. A cross section through the original coarse-scale trajectory segment is a Cantor Set as illustrated below. At any particular level of magnification (i.e., fractal iterate), the trajectory segments can be labelled *a* or a according to the regime to which they evolve.

**Figure 2:**
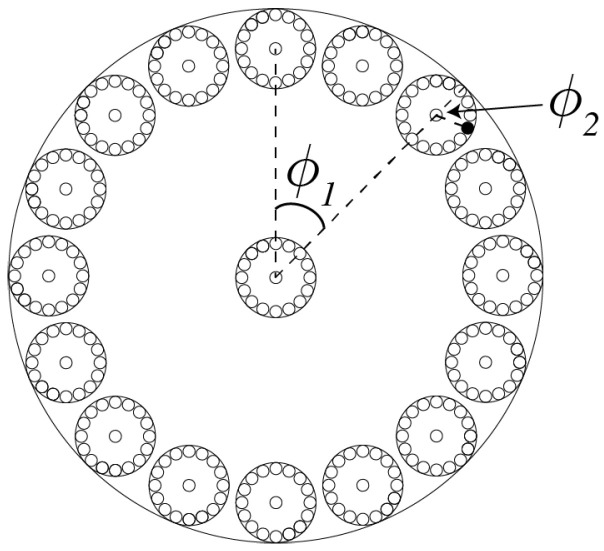
A Cantor Set C, comprising p=17 iterated disks: N=16 iterated pieces around the edge of a disk and 1 at the centre of a disk. Here, a single disk at the (j−1)th fractal iteration comprises 17 *j*th-iterate disks, and each of these comprises 17 (j+1)th-iterate disks. An element of C can be represented by a sequence $\left\{\phi_{1},\phi_{2},\phi_{3},\ldots \right\}$, where $\phi_{i}/2\pi =n/N\in Q$.

Results below summarise more detailed analysis given in [6]. As mentioned above, we posit some primal compact fractal-like geometry $I_{U}$ in cosmological state space, on which the universe *U* as a self-contained locally causal deterministic system evolves [4,5]. Figure 1 illustrates the local fractal structure of $I_{U}$. On the left is shown, at some (j−1)th fractal iterate of $I_{U}$, a single state-space trajectory segment (‘history’) in some three-dimensional subspace of state space. At the *j*th iterate, this trajectory segment comprises a helix of N≫0 fine-scale trajectories and an additional N+1th trajectory (not shown) at the centre of the helix. The winding frequency ω of a *j*th iterate helical segment is assumed proportional to the energy *E* associated with the subsystem described by this subspace. In this sense, the deBroglie relationship E=ℏω reflects a key element of the invariant set postulate: that the laws of physics in space-time are manifestations of the geometry of the more primal $I_{U}$. At the (j+1)th iterate (not shown), the helical trajectory segments are themselves found to be helical. In general, a cross section through a (j−1)th trajectory segment comprises a Cantor set C comprising p=N+1 iterated disks (Figure 2).

**Figure 3:**
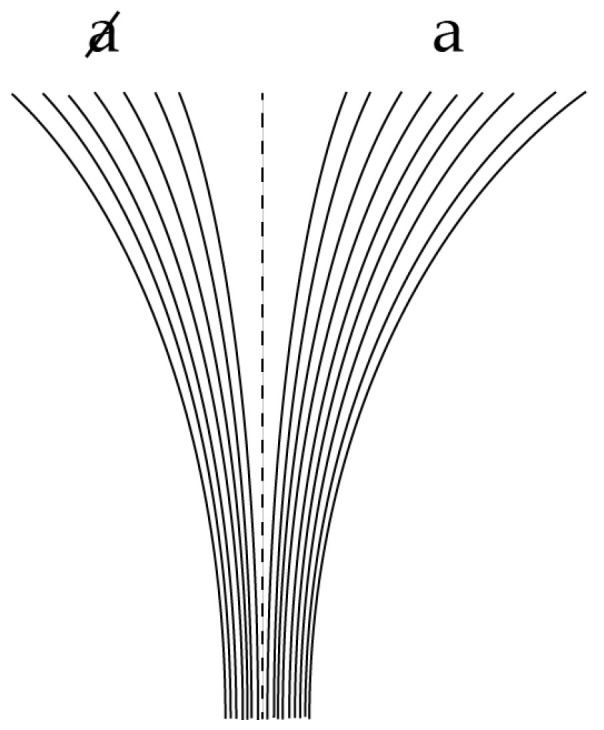
Here, N=16 classical state-space trajectories diverge into two distinct regimes labelled *a* and a. In this example, seven of the 16 evolve to the a regime and the other nine evolve to the *a* regime. In terms of the parameter θ described in the text, here cosθ=1/8∈Q.

We will now suppose that, at any fractal iterate, the *N* helical trajectory segments in Figure 1 can be labelled according to a process illustrated in Figure 3, associated with the divergence and nonlinear clustering of trajectories into two distinct state-space regimes or clusters labelled *a* and a (the central (N+1)th trajectory is assumed to lie on the basin boundary and hence to evolve neither to *a* nor a). This divergence reflects the generic phenomenon of decoherence (essentially the butterfly effect) as the sub-system interacts with its environment. These clusters correspond to the measurement eigenstates of quantum theory.

Within this geometric framework, complex Hilbert vectors can be used to provide some incomplete probabilistic description of reality. For example, consider the (j+1)th iterate disks inside a *j*th iterate disk and labelled $\phi_{2}$ in Figure 2. Suppose that $N\cos^{2}\left(\theta /2\right)$ of these (j+1)th iterate disks are labelled *a*, and that *reality* corresponds to one of the *N* disks and is therefore either labelled *a* or a. Then, as discussed in [6] in more detail and with $\phi =\phi_{2}$, an incomplete representation of *reality* can be given probabilistically by the complex Hilbert vector

(3) cosθ2|a〉+eiϕsinθ2|a〉.

In particular, it is necessary that ϕ/2π and $\cos^{2}\left(\theta /2\right)$ (and hence cosθ) are rational numbers. By contrast, a putative Hilbert vector where cosθ∉Q or ϕ/2π∉Q cannot provide an incomplete representation of any trajectory segment on $I_{U}$ and, therefore, in Invariant Set Theory, cannot correspond to an ontic state. More general tensor-product Hilbert states can also be used to provide incomplete representations of multi-variate properties of *reality*. Again, it is necessary that all squared amplitudes are rational, and all complex phase angles are rational multiples of 2π [6].

A crucial number theorem that completely underpins this framework is the following:

**Theorem** **1.** *Let ϕ/π∈Q. Then, cosϕ∉Q except when $\cos\phi =0,\pm \frac{1}{2},\pm 1$. [9,10]*

**Proof.**  Assume that 2cosϕ=a/b, where a,b∈Z have no common factors and b≠0. Since $2\cos2\phi ={\left(2\cos\phi \right)}^{2}-2$, then $2\cos2\phi =\left(a^{2}-2b^{2}\right)/b^{2}$. Now, $a^{2}-2b^{2}$ and $b^{2}$ have no common factors, since if *p* were a prime number dividing both, then $p|b^{2}\Rightarrow p|b$ and $p|(a^{2}-2b^{2})\Rightarrow p|a$, a contradiction. Hence, if b≠±1, then the denominators in 2cosϕ,2cos2ϕ,2cos4ϕ,2cos8ϕ⋯ get bigger without limit. On the other hand, if ϕ/π=m/n, where m,n∈Z have no common factors, then the sequence ${\left(2\cos2^{k}\phi \right)}_{k\in N}$ admits at most *n* values. Hence, we have a contradiction. Hence, b=±1 and $\cos\phi =0,\pm \frac{1}{2},\pm 1$. ☐

We now define a metric $g_{p}$ that respects the primacy of $I_{U}$ where ontic states on $I_{U}$ and non-ontic states off $I_{U}$ are necessarily distant from one another (no matter how close they may appear from a Euclidean perspective). For all x∈C, y∈C and z∉C,

- $g_{p}\left(x,y\right)$ is Euclidean,
- $g_{p}\left(x,y\right)\leq 1$,
- $g_{p}\left(x,z\right)=g_{p}\left(y,z\right)=p.$

Hence, if p≫1, we can say that *z* is $g_{p}$-distant from both *x* and *y*. It is easily shown that $g_{p}$ satisfies the axioms for a metric (e.g., the triangle inequality) on cosmological state space and that $g_{p}$ is related to the *p*-adic metric of number theory [6].

## 3. The Sequential Stern-Gerlach Experiment

**Figure 4:**
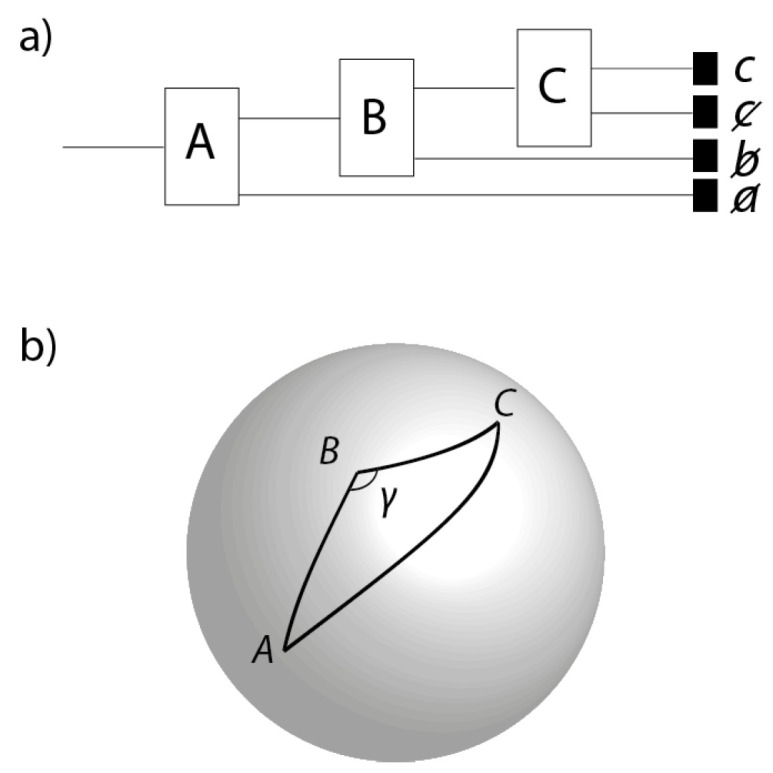
(**a**) a sequential Stern-Gerlach experiment where a particle is sent through three Stern-Gerlach devices, A, B and C; (**b**) A, B and C shown as directions on the celestial sphere. Although to experimental accuracy A, B and C may be coplanar, they are not coplanar precisely. In invariant set theory, we demonstrate the non-commutativity of spin observables by number theory.

As a warm up to the Bell Theorem, we discuss in this Section one of the classic experiments designed to introduce students to non-commutativity of spin observables in quantum theory (e.g., [11]). Consider an ensemble of spin-1/2 particles prepared by the first of three Stern-Gerlach apparatuses (Figure 4a) with spins oriented in the direction $\hat{a}$ in physical 3-space. The particles that are prepared spin-up by this first apparatus pass through a second Stern-Gerlach apparatus oriented in the direction $\hat{b}$. The particles that are output along the spin-up channel of the second apparatus are then passed into a third Stern-Gerlach apparatus oriented in the direction $\hat{c}$. The directions $\hat{a}$, $\hat{b}$ and $\hat{c}$ correspond to points *A*, *B* and *C* on the celestial sphere $S^{2}$ (Figure 4b). Typically, the directions $\hat{a}$, $\hat{b}$ and $\hat{c}$ are designed to be coplanar, i.e., *A*, *B* and *C* lie on a great circle. However, this is impossible to achieve *precisely*: as a matter of principle, one cannot shield the experiment from the distorting effects of gravitational waves. Hence, as in Figure 4b, we assume that *A*, *B* and *C* are the vertices of some non-degenerate triangle ▵ABC, where the angle γ is not equal to $180^{\circ }$ precisely.

We now show that if a particle was measured by the apparatus in the order A-B-C, then it could not have been measured in the order A-C-B. That is, the measurements in directions $\hat{b}$ and $\hat{c}$ cannot be performed simultaneously. In Invariant Set Theory, this result is derived by number theory. Consider a particle sent through the sequential Stern-Gerlach apparatus in the configuration A-B-C and where the detector corresponding to either *c* or c was triggered. Then, in Invariant Set Theory, we require that all of $\cos\theta_{AB}$, $\cos\theta_{BC}$ and γ must be rational for the experiment to lie on $I_{U}$ and hence correspond to *reality*. We now ask the question: what would the outcome have been for that particle had the configuration been A-C-B. For there to have been a definite outcome, we require, in addition, that $\cos\theta_{AC}\in Q$. However, by the cosine rule for spherical triangles

(4) $$\cos\theta_{AC}=\cos\theta_{AB}\cos\theta_{BC}+\sin\theta_{AB}\sin\theta_{BC}\cos\gamma .$$

The right-hand side is the sum of two terms. The first is rational since it is the product of two terms each of which, by construction, is rational. The second is the product of three terms the last of which, cosγ, is irrational, except for the eight exceptions listed in the Theorem above. Since γ is only approximately equal to $180^{\circ }$, cosγ is irrational. Since $\theta_{AB}$, $\theta_{BC}$ and γ are independent degrees of freedom defining the triangle ▵ABC, there is no reason why $\sin\theta_{AB}$ and $\sin\theta_{BC}$ should conspire with cosγ to make the product $\sin\theta_{AB}\sin\theta_{BC}\cos\gamma$ rational. Hence, $\cos\theta_{AC}$ is the sum of a rational and an irrational and is therefore irrational. Hence, for the particle where A-B-C was measured, the counterfactual A-C-B is undefined and could not be an element of *reality*. Put another way, the state $U^{\prime }\notin I_{U}$ of the universe associated with the configuration A-C-B is $g_{p}$ distant from the state $U\in I_{U}$ associated with configuration A-B-C.

We can of course envisage performing two separate sequential Stern-Gerlach experiments (one on a Monday, the other on a Tuesday, say) where the order of the Stern-Gerlach apparatuses was A-B-C and A-C-B, respectively. For Monday’s experiment, $\cos\theta_{AB}$ and $\cos\theta_{BC}$ are rational, and the angle subtended at *B* is a rational multiple of 2π. For Tuesday’s experiment, $\cos\theta_{AC}$ and $\cos\theta_{BC}$ are rational, and the angle subtended at *C* is a rational multiple of 2π. As before, this would be impossible if the triangle ▵ABC was *precisely* the same on Monday and Tuesday. However, this will not be the case—background space-time ripples are necessarily different on Tuesday compared with Monday.

One potential objection should be answered before moving on to the Bell Theorem. In the discussion above, θ denoted a relative orientation in physical space, whereas in the discussion in Section 2, θ was merely a parameter whose cosine gave the probability of one measurement outcome rather than another. How is it that θ can now be interpreted as an orientation in physical space? The answer relates to the existence of spinorial structure on C. To see this, the reader is directed to [6].

## 4. The Bell Inequality

**Figure 5:**
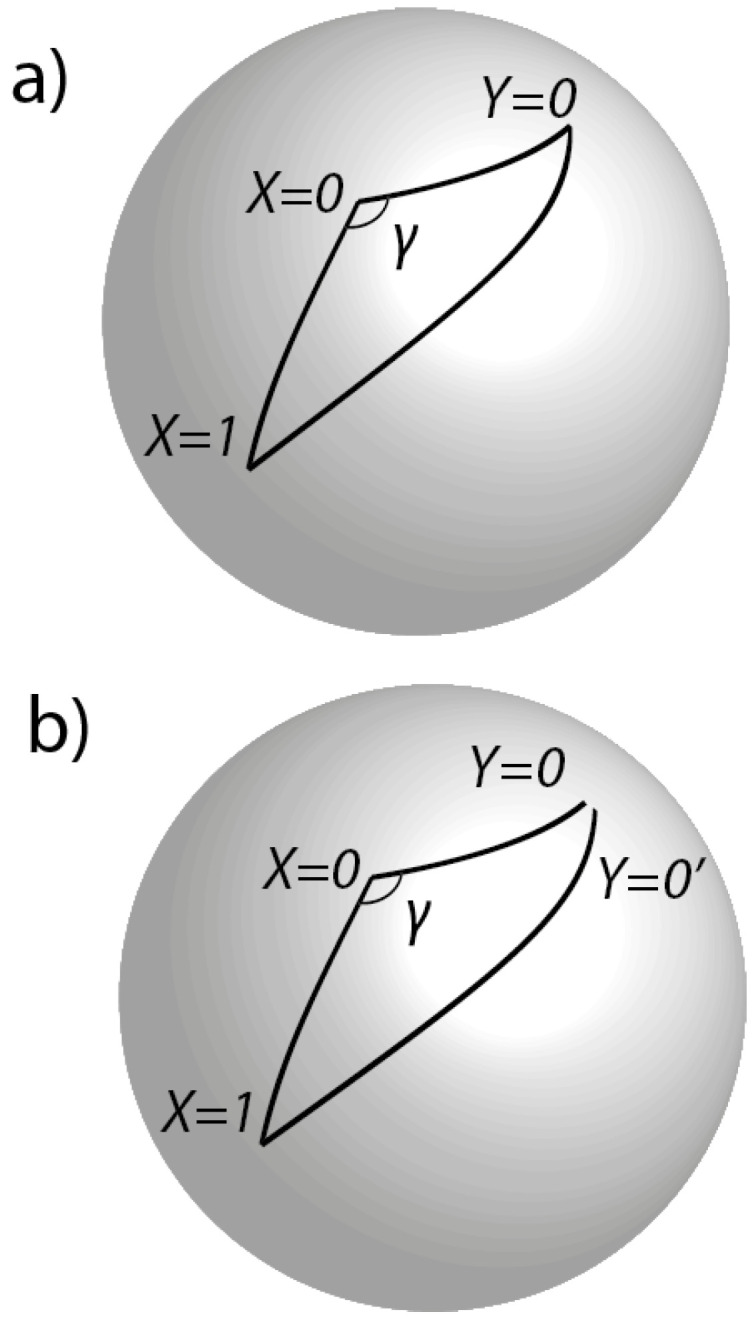
(**a**) in general, it is impossible for all the cosines of the angular lengths of all three sides of the spherical triangle to be rational, and the internal angles rational multiples of 2π. (**b**) what actually occurs when (2) is tested experimentally. Here, the cosines of the angular lengths of all sides are rational. In a precise sense, (b) is $g_{p}$ distant from (a).

Consider now the relationship between (1) and (2) from the perspective of Invariant Set Theory. As above, let X=0,1, Y=0,1 denote four random points on the sphere, three of which (relevant to the discussion below) are shown in Figure 5a. Let $\theta_{XY}$ denote the relative orientation between an *X* point and a *Y* point. Recall that complex Hilbert states can represent uncertain trajectory segments on $I_{U}$ providing squared amplitudes are rational. Hence, $\mathrm{Corr}\left(X,Y\right)=-\cos\theta_{XY}$ requires $\cos\theta_{XY}\in Q$.

Suppose Alice freely chooses X=0 and Bob Y=0 when measuring a particular entangled particle pair. Then, it must be the case that $\cos\theta_{00}\in Q$. Could Alice and Bob have chosen X=1 and Y=0 when measuring this same particle pair, given that they actually chose X=0, Y=0? In other words, does the state $U^{\prime }$ of the universe in which this counterfactual experiment takes place also lie on $I_{U}$? To answer the question in the affirmative, we additionally require $\cos\theta_{10}\in Q$. However, as with the Stern-Gerlach analysis, applying the cosine rule for spherical triangles, we have

(5) $$\cos\theta_{10}=\cos\theta_{00}\cos\alpha_{X}+\sin\theta_{00}\sin\alpha_{X}\cos\gamma ,$$

where $\alpha_{X}$ is the angular distance between X=0 and X=1. Now, it is always possible for Alice to send the particle which she has just measured in the X=0 direction, back into her measuring apparatus to be again measured in the X=1 direction. Hence, $\cos\alpha_{X}$ must be rational. Now, we also require the angle γ to be a rational multiples of 2π. This would be so if the three points X=0,1 and Y=0 lay on a great circle *exactly*, so that $\gamma =180^{\circ }$*precisely*. However, as before, because of ubiquitous unshieldable gravitational waves, this cannot be the case. Hence, $\cos\theta_{01}$ is the sum of two terms, the first a rational and the second the product of three independent terms, the last of which is irrational. Being independent, these three terms cannot conspire to make their product rational. Hence, $\cos\theta_{01}$ is the sum of a rational and an irrational and must therefore be irrational. Hence, the state of the universe $U^{\prime }$ in which the counterfactual experiment takes place is not realistic and is $g_{p}$-distant from worlds on $I_{U}$. Hence, the counterfactual question cannot be answered in the affirmative: Corr(1,0) is undefined. In general, it is never the case that all four correlations in (1) are definable on $I_{U}$—the Bell inequality is always undefined in Invariant Set Theory.

An experimenter might ask how one could set up an experiment with sufficient care to ensure that the corresponding Hilbert state descriptors were rational rather than irrational. The answer is that the experimenter need take no care: if an experiment is performable, i.e., corresponds to some $U\in I_{U}$, then by construction the descriptors must be rational. Physical perturbations (e.g., gravitational waves in space-time) only introduce uncertainty in the values of the rational descriptors and not in the fact that they are rational. Conversely, if the descriptor of a counterfactual state is irrational, then no amount of noise that respects the primacy of $I_{U}$ can change it into an ontic state. This property provides an attractive finitist feature that is missing in conventional physical theories based on R or C and hence in theories that utilise the Euclidean state-space metric. Hence, in the real world of experiments, both $\cos\theta_{00}$ and $\cos\theta_{10^{\prime }}$ in (2) are necessarily and robustly rational (Figure 5b), consistent with the fact that the individual sub-ensembles are measured at different times and/or locations, and that unshieldable gravitational waves ensure that orientations are not *precisely* the same when these different sub-ensembles are measured. Indeed, we can infer the existence of an effectively infinite family of orientations where all of $\cos\theta_{00},\cos\theta_{10^{\prime }},\cos\theta_{0^{\prime }1},\cos\theta_{1^{\prime }1^{\prime }}$ in (2) are rational. However, by construction, none of the orientations so generated includes those associated with (1), which is therefore indeed the singular limit of and $g_{p}$-distant from (2). Just as the paradox of the Penrose ‘impossible triangle’ is resolved by realising that the sides of the triangle are not necessarily close near a vertex of the triangle, so too here. As discussed in [6], many of the familiar ‘paradoxes’ of quantum theory can be interpreted realistically and causally with $g_{p}$ as the metric of state space.

## 5. Conspiracy and Free Will

As discussed, if the spins of an entangled particle pair are measured relative to X=0, Y=0, then by construction the spins of this particular particle pair could not have been measured relative to the directions X=1 and X=0, respectively. This is nothing to do with entanglement per se, but is rather a manifestation of quantum complementarity (associated with the non-commutativity of quantum observables). For example, as discussed in Section 3, if an experimenter performs a sequential Stern-Gerlach experiment in the order A-B-C, then, as a matter of principle, an experiment could not have been performed on the same particle in the order A-C-B. That is to say, if the state *U* of the universe associated with one experiment lies on $I_{U}$, then the corresponding state $U^{\prime }$ of the universe associated with the other experiment does not lie on $I_{U}$ and is $g_{p}$ distant from states on $I_{U}$.

This implies a violation of the principle of Measurement Independence (MI), usually framed in terms of some probability density ρ(λ) of so-called ‘hidden-variables’ λ associated with the particles in question. In particular, MI is violated if

(6) ρ(λ)≠ρ(λ|m),

where *m* denotes some particular measurement, e.g., the A-B-C experiment or the X=0, Y=0 experiment. Another way of saying this is that the ‘hidden variables’ are contextual [12] and it is well known that contextual hidden variables could provide a route to negating the Bell Theorem. However, violation of MI is often seen as either implausibly conspiratorial [13] or inconsistent with experimenter free will [14]. As discussed below, Invariant Set Theory is neither implausibly conspiratorial nor inconsistent with experimenter free will.

It is important in the discussion below to recognise that, in Invariant Set Theory, the violation of MI is not imposed by fiat. Rather, it is a consequence of the postulate that $I_{U}$ is a primal fractal-like geometry on which states of the universe evolve.

### 5.1. Nullifying the Notion of Conspiracy

Consider a specific and pertinent example. Suppose, in a Bell experiment, the measuring apparatuses are set according to the frequency ν of photons emitted by distant stars. If $\nu <\nu_{0}$, a reference frequency, suppose the X=0, Y=0 directions are chosen; if $\nu >\nu_{0}$, then the X=1, Y=0 directions are chosen. Let $\Lambda_{00}$ denote some sample space of hidden variables associated with the choice X=0, Y=0 and so on. In Invariant Set Theory, if $\lambda \in \Lambda_{00}$, then the outcome of measurements in the X=0, Y=0 directions is well defined, but, by the discussion above, the outcome of measurements in the X=1, Y=0 directions is undefined. Conversely, if $\lambda \in \Lambda_{10}$, then the outcome of measurements in the X=1, Y=0 directions is well defined, but the outcome of measurements in the X=0, Y=0 directions is undefined.

The notion of conspiracy arises because we have two seemingly independent pieces of information that determine the type of measurement made: the frequency ν and the hidden variables λ. Since the photon is emitted long before the entangled particles, and in a different part of the universe, one could imagine that these quantities can be varied independently of one another. If this is so, then the possibility that $\nu <\nu_{0}$ but $\lambda \in \Lambda_{10}$ leads to inconsistency since the latter combination is associated with a state of the universe not lying on $I_{U}$. Hence, there must be some unpalatable ‘conspiracy’ between ν and λ, so the argument goes, to prevent such inconsistency.

However, this conclusion is incorrect. Firstly, a thousand years (say) before the experiment is performed, the photon does not and cannot ‘know’ that its frequency (and not, say, bits from a to-be-made sci-fi movie) will be the determinant of some future measurement settings. If the experimenters decide what whimsical process (photons or movie bits) they will use to set the measurement orientations at space-time event *D*, then the information needed to determine the nature of this decision will be completely delocalised on spacelike hypersurfaces in the causal past of *D*. Perturbing any one bit anywhere in the causal past of *D* could change the nature of this decision. This is the butterfly effect, generic to nonlinear systems evolving on fractal attractors, and the stuff of numerous sci-fi movies.

Similarly, it is incorrect to assume that the entangled particles ‘know’ whether their hidden variables belong to $\Lambda_{00}$ or $\Lambda_{10}$. Consistent with both the quantum field-theoretic notion of a particle as a field excitation, and the invariant set premise that the laws of physics in space-time derive from a holistic geometry in state space, it is incorrect to think of λ as somehow internal and localised to the particles being measured. Consider the following analogy. Babies (almost always) either belong to the set $\Lambda_{\mathrm{male}}$ or $\Lambda_{\mathrm{female}}$ of babies who are male or female at birth. Information that determines which of the two sets a particular baby belongs is internal to the baby. This type of analogy describes classical hidden-variable theory (probe the particle to find its hidden variables and determine what spin it will have when measured in a particular direction), but does not describe the situation here. A more accurate analogy is this: at birth, all babies either belong to the set $\Lambda_{L}$ or ΛL of humans that, as adults, either fall in love or don’t. Information that determines to which of $\Lambda_{L}$ or ΛL a particular baby belongs is clearly not internal to the baby. In particular, if $L_{B}$ denotes the event where adult Bob falls in love, then the information λ that determines that baby Bob belongs to $\Lambda_{L_{B}}$ is (as above) completely delocalised on spacelike hypersurfaces in the causal past of $L_{B}$ and hence cannot, in principle, be known to baby Bob. Because λ is delocalised in this way, then when a counterfactual experiment is described as an alternative experiment on the same particle, i.e., on the same λ, effectively we are describing a hypothetical experiment where the measurement set up is changed, but the rest of the universe is held fixed.

In short, there is no implausible conspiracy between the so-called hidden variables and determinants of the processes which set the measurement observations. As discussed, information which determines these supposedly independent quantities are in fact highly intertwined on spacelike hypersurfaces in the causal past of the experiment.

### 5.2. Free Will and Inaccessible Determinism

The only way there can be a conflict between free will and determinism is if it was found to be possible to compute the future algorithmically, faster than the universe actually evolves. If a computer can predict my future actions reliably, then I am an automaton. Without this, determinism and free will are completely compatible with one another. Is it possible to compute the future with some faster-than-reality computational subset of the universe? No! If $I_{U}$ was a strict fractal, then it would actually be non-computational [15,16]. However, if $I_{U}$ is some finite fractal-like limit cycle, it will still have a property called computational irreducibility [17]: we cannot reliably predict which set of measurement settings will be chosen with a computationally simpler approximation of the full system. In particular, supressing just one bit of information on some initial spacelike hypersurface when integrating forward in time can lead to a completely different choice of measurement setting. This is again the butterfly effect and is generic for systems that evolve on fractal invariant sets in state space. This property of computational irreducibility can be considered as implying an ‘inaccessible’ form of determinism.

Sometimes the word ‘pre-destination’ is used as a synonym for determinism. For example, in a deterministic world, it was already pre-destined at the time of the dinosaurs that Alice would do this measurement and not that. For some, such pre-destination sounds implausible. What is sometimes forgotten with this example is that the information that determines that Alice would do this measurement and not that at the time of the dinosaurs is completely delocalised on the intersection of some spacelike hypersurface at the time of the dinosaurs with the causal past of the event, where Alice makes the measurement. That is to say, the information that determines Alice’s measurement choice is completely inaccessible at the time of the dinosaurs: it is buried down at the Planck scale over regions of space spanning hundreds of millions of light years. Changing just one bit on a Planck-scale variable on this hypersurface could change what measurement Alice makes. For the third time, this is the butterfly effect and generic for systems that evolve on fractal invariant sets. Is that problematic for deterministic theories of physics?

Further discussion of the free-will issue is given in Section 7.

## 6. Relations to Other Approaches

In this section, we discuss the relationship between Invariant Set Theory and some other approaches to quantum physics.

### 6.1. Bohmian Theory

Since this paper was written as part of a celebration of the 100th birthday of David Bohm, it is worth commenting on possible links to the de-Broglie/Bohm interpretation of quantum theory [18]. Both Bohmian theory and Invariant Set theory are deterministic. However, Bohmian theory is necessarily non-local (i.e., not locally causal) whilst Invariant Set theory is not. The reason for this difference hinges around differences between the quantum potential, a differentiable potential function in configuration space, and $I_{U}$ a fractal geometric object in state space. In particular, because there are no ‘holes’ in the quantum potential, Bohmian theory must be counterfactually complete and hence satisfy MI (and can therefore only violate the Bell Theorem by being nonlocal). By contrast, as discussed, the Invariant Set does generically have holes, is therefore not counterfactually complete, and hence does not satisfy MI and therefore is not required to satisfy the Bell inequalities despite being locally causal.

This raises the following tantalising possibility. Perhaps one should think of the Bohmian quantum potential as a ‘coarse-grained’ approximation to the fractal geometric structure of state space. As a simple illustration, it would be possible to mimic some aspects of the behaviour of the state vector of the Lorenz ’63 system [19] by a stochastically forced motion in a double potential well. Of course, the fractal structure of the attractor would be ‘smoothed out’ in such a potential-well system. Thinking of the quantum potential as an approximation to some object in state space with rich geometric number-theoretic structure may provide a direction in which to move Bohmian theory forward.

### 6.2. The Cellular Automaton Interpretation of Quantum Mechanics

In both Invariant Set Theory and the Cellular Automaton Interpretation of Quantum Mechanics [20], it is concluded that quantum physics can be described by deterministic causal laws, and where the Bell Theorem is negated through a failure of counterfactual incompleteness [20]. However, the reasons for rejecting counterfactual completeness are different in these two approaches. The Cellular Automaton Interpretation rejects counterfactual definiteness by the assumption that the cosmological initial conditions are somehow special, in the sense that counterfactual perturbations to the initial conditions that would lead to the counterfactual measurements, are somehow excluded from the theory. In common with contemporary physics, ’t Hooft separates the laws of physics from the initial conditions, i.e., treats them as separate. Treating the initial conditions as special in this way could be viewed as ad hoc, as well as being imprecise. What exactly is it about the initial conditions that prevents these quantum counterfactuals?

By contrast, in Invariant Set Theory, the initial conditions and the laws of physics are not independently specifiable items and that the Universe as a dynamical system evolves on the invariant set. Hence, by constructions, the cosmological initial conditions must lie on the invariant set. That is to say, there is no fundamental distinction between the laws of physics and the initial conditions in the sense that there is in standard theory. It is the structure of the invariant set that leads to counterfactual incompleteness i.e., where certain quantum counterfactual perturbations take a state lying on the invariant set and take it off the invariant set. Ultimately, the structure that leads to this property is number theoretic: that rational angles cannot have rational cosines, and vice versa.

### 6.3. p-Adic Quantum Theory

Over the years, there have been attempts to reformulate quantum theory, e.g., by replacing the complex coefficients of Hilbert states with *p*-adic numbers (there is a *p*-adic correspondence) to the complex numbers. A review of such approaches is given in [21]. One of the motivations for this was the idea that space-time may have some fractal structure on the Planck scale. Drawing on this work, Khrennikov [22] proposed a hidden-variable model where the probability distribution ρ(λ) was defined on the *p*-adic numbers. Now, essentially because −1=1+2+4… in 2-adic number theory, this allows for negative probabilities, which, although very esoteric, also arose for different reasons in Dirac’s work on relativistic quantisation, and therefore have some basis in quantum physics. Khrennikov argued that the existence of such negative probabilities could negate the Bell Theorem.

It is important to distance the present work from such *p*-adic quantum theory. Here, we do not introduce *p*-adic numbers into Hilbert Space or (as a result) into probability distributions. Fundamentally, Invariant Set Theory is a deterministic theory and not a probabilistic theory. A key element of Invariant Set Theory is to reject the notion of the algebraically closed Hilbert Space as a state space for quantum physics, irrespective of whether the Hilbert Space is closed on the reals, the complexes or the *p*-adics. The reason for such rejection of this is ultimately so that physics can be described finitely [6]. In Invariant Set Theory, probabilities are defined by elementary frequentism and therefore are simply and intuitively rational numbers on the interval [0,1]. In fact, in the work presented in [6], maps on *p*-adic integers are defined to describe the deterministic laws that may underpin quantum physics. This is where algebraic closure can be reinstated—not on the Hilbert vectors or associated probabilities.

Indeed, the metric $g_{p}$ that plays such a central role in this paper, whilst motivated by the *p*-adic metric and the fact that the set of *p*-adic integers is homeomorphic to Cantor sets, is not identical to it. $g_{p}$ has the property that, if *p* is large, points which do not lie on $I_{U}$ are necessarily distant from points that do lie on $I_{U}$. This reflects the fact that the *p*-adic distance between *p*-adic numbers that are not *p*-adic integers, and *p*-adic integers, is ≥p.

This comparison with *p*-adic quantum theory raises an important point. A key motivation for developing Invariant Set Theory (in addition to be able to describe physics finitely) was to make the Bell Theorem understandable. If a particular formalism leads one to trade one type of incomprehensible property (nonlocality) for another (negative probability), then, as far as the author is concerned, there is no compelling reason to adopt such a formalism. As discussed further in the next Section, Invariant Set Theory does not require any esoteric notions to explain the Bell Theorem (once one has embraced the key fact that space-time and state-space can have very different metrics).

## 7. Discussion

A theoretical framework has been outlined that asserts that no physical experiment has or will demonstrate that the Bell inequality (1) is violated—even approximately. In this framework, (1) is the singular limit of the experimentally tested (2), (1) is undefined and (2) is not approximately equivalent to (1). Key to this formulation, a non-Euclidean metric $g_{p}$ is introduced on state space. $g_{p}$, related (but not entirely equivalent) to the *p*-adic metric of number theory, respects the primacy of an assumed fractal geometry $I_{U}$ on which the universe is assumed to evolve and from which the laws of physics derive. Based on $g_{p}$, we can make an ontological distinction between Hilbert vectors with rational descriptors (rational squared amplitudes and rational complex phases) and irrational descriptors. Based on this framework, it is claimed that experiments do not rule out Einsteinian determinism and causality. Only in the singular classical limit at p=∞ could experiments be used to rule out local causality.

Making an ontological distinction between Hilbert vectors with rational and irrational descriptors is likely to induce a sense of unease (indeed scepticism) amongst many readers; not least, the results above may appear to be inconsistent with the experimental fact that the violation of Bell-like inequalities is insensitive to the precise orientation of polarisers. To alleviate this sense of unease, consider the function f(x) on [0,1] such that $f\left(x\right)=x^{2}$ if *x* is rational, and f(x)=3 (say), otherwise. This function is everywhere discontinuous and hence non-differentiable on the reals, and therefore f(x) could hardly describe how experimental values vary smoothly with experimental parameter *x*. However, consider a physical theory *T* that demands that states of reality (that is to say, states of systems that can be probed by experiment or are otherwise amenable to observation) are only associated with rational values *x* and that irrational only arise in *T* when considering hypothetical counterfactual states that did not occur in reality. To have such a property, *T* would be a profoundly nonlinear theory. Based on this, *T* has the property that f(x) is not only continuous but also (using the rational calculus) differentiable over the set of physically realistic values of *x*. Over a large number of parameter values *x*, an experimenter might return the values $f\left(x\right)=x^{2}+\epsilon$, where ϵ denoted some random experimental error. The experiments would, by construction, never return the value f(x)=3. As a result, a theoretician (unaware of *T*) might construct a linear theory $T^{\prime }$ on the reals where $f\left(x\right)=x^{2}$ for all 0≤x≤1. Like *T*, $T^{\prime }$ would describe the results of experiments well. Being linear, $T^{\prime }$ would be analytically and computationally tractable, making it a convenient tool in practice. However, $T^{\prime }$ would incorrectly ascribe values to counterfactual states and this could lead to inconsistencies in the interpretation of $T^{\prime }$. Some might argue that since, by construction, such inconsistencies have no implications for the real world of experimentation, one should ‘shut up and calculate’ with $T^{\prime }$ and not waste time searching for the deeper theory *T*. However, the failure of $T^{\prime }$ to describe the nonlinear structure in *T* may have implications elsewhere, e.g., when trying to extend $T^{\prime }$ to account for phenomena beyond the experiments that have so far been conducted (see below).

However, unlike $T^{\prime }$, *T* is an unrealistically fine-tuned theory [23,24], since rationals and irrationals lie arbitrarily close to each other on the real line with respect to the standard Euclidean metric. The notion that distances in physics should be necessarily described by the Euclidean metric is a deeply held intuition, since almost the first thing we learn as babies is a sense of spatial awareness (for the baby to get its hand close to a colourful toy, it has to learn to equate closeness with smallness of Euclidean distance). However, here we are considering distances in state space, not in space time, and where our intuitions may not apply. This raises the question about whether there is a state-space metric where realistic and counterfactual states are actually distant from one another. The toy model example here is too simple to allow such an alternate interpretation. However, using fractal geometry we have shown that there is a model where such states are indeed distant from one another, thus negating the fine-tuning argument.

As discussed in the Introduction, a rather beautiful example of how relying on intuition about distance can lead to inconsistency is provided by the Penrose Impossible Triangle. We claim that quantum theory (cf., $T^{\prime }$) is similarly inconsistent, even though it is wonderfully accurate and a convenient tool for analytic manipulation and computation. This inconsistency arises from the use of the Euclidean metric forced on us by the assumption that state space is the algebraically closed Hilbert Space. By weakening this assumption, allowing only Hilbert states with rational descriptors as elements of physical reality, the inconsistencies associated with the Bell and other no-go theorems [6], disappear. The key conclusion we can draw from this discussion is that, in Invariant Set Theory, there is no contradiction with the fact that the violations of (2) are insensitive to polariser orientation.

Let us now discuss a related issue. Let us fix the orientation of Bob’s measuring device and ask whether, according to Invariant Set Theory, this in any way constrains Alice in orienting her measuring device. If Alice was somehow constrained, she would not only not be a free agent, she would somehow be remotely under the influence of Bob—clearly unacceptable in any theoretical framework purporting to reinstate the notion of Einsteinian local realism. The answer is that, for all practical purposes, Alice is under no such constraint. What ‘for all practical purposes’ means is that within *any* neighbourhood of Alice’s celestial sphere, no matter how small, there exist orientations which Alice is free to choose from, providing *p* is sufficiently big. That is to say, the set of orientations from which Alice can choose is as dense as one likes, providing *p* is big enough. Colloquially, we can indeed say that Alice can set her measuring apparatus as she pleases—she is a free agent in any practical sense of the phrase. Nevertheless, whatever the size of *p*, Invariant Set Theory requires that the cosine of the relative orientation between Alice and Bob’s measuring apparatuses must be rational. For large *p*, this is an utter irrelevance in the *design* of a Bell experiment. However, it is crucially important in the *interpretation* of a Bell experiment, since, as discussed, the counterfactual states needed to establish (1) will inevitably lie off $I_{U}$ and are therefore $g_{p}$ distant from the states measured by experiment. In conclusion, in making the statement that the orientation of Bob’s polariser does not influence Alice’s choice of polariser orientation, the contrast between the Euclidean metric of space-time and the metric $g_{p}$ of state space becomes crucial.

The author believes that the ongoing failure to synthesise quantum and gravitational physics satisfactorily arises from the fact that quantum theory is inimical to the local realism of general relativity, and a synthesis between these two areas of physics will require a nonlinear theory of quantum physics, less like $T^{\prime }$ and more like *T*. As such, in the analysis above, it is plausible that $p^{-1}$ defines the gravitational coupling constant (and so the largeness of *p* reflects the weakness of gravity). Indeed, the fact that gravitational waves provide an *in principle* unshieldable source of noise to ensure that rational angles can never have rational cosines (a central theorem to this paper) may be evidence of a deep link to the phenomenon of gravity, with experimental consequences for the dark universe and for quantum gravity. These more speculative notions have been developed elsewhere [25].
